# Cathepsin Gene Family Reveals Transcriptome Patterns Related to the Infective Stages of the Salmon Louse *Caligus rogercresseyi*

**DOI:** 10.1371/journal.pone.0123954

**Published:** 2015-04-29

**Authors:** Waleska Maldonado-Aguayo, Jacqueline Chávez-Mardones, Ana Teresa Gonçalves, Cristian Gallardo-Escárate

**Affiliations:** Laboratory of Biotechnology and Aquatic Genomics, Interdisciplinary Center for Sustainable Aquaculture Research (INCAR), University of Concepción, Concepción, Chile; University of Heidelberg, GERMANY

## Abstract

Cathepsins are proteases involved in the ability of parasites to overcome and/or modulate host defenses so as to complete their own lifecycle. However, the mechanisms underlying this ability of cathepsins are still poorly understood. One excellent model for identifying and exploring the molecular functions of cathepsins is the marine ectoparasitic copepod *Caligus rogercresseyi* that currently affects the Chilean salmon industry. Using high-throughput transcriptome sequencing, 56 cathepsin-like sequences were found distributed in five cysteine protease groups (B, F, L, Z, and S) as well as in an aspartic protease group (D). Ontogenic transcriptome analysis evidenced that L cathepsins were the most abundant during the lifecycle, while cathepsins B and K were mostly expressed in the larval stages and adult females, thus suggesting participation in the molting processes and embryonic development, respectively. Interestingly, a variety of cathepsins from groups Z, L, D, B, K, and S were upregulated in the infective stage of copepodid, corroborating the complexity of the processes involved in the parasitic success of this copepod. Putative functional roles of cathepsins were conjectured based on the differential expressions found and on roles previously described in other phylogenetically related species. Moreover, 140 single nucleotide polymorphisms (SNP) were identified in transcripts annotated for cysteine and aspartic proteases located into untranslated regions, or the coding region. This study reports for the first time the presence of cathepsin-like genes and differential expressions throughout a copepod lifecycle. The identification of cathepsins together with functional validations represents a valuable strategy for pinpointing target molecules that could be used in the development of new delousing drugs or vaccines against *C*. *rogercresseyi*.

## Introduction

*Caligus rogercresseyi*, like the European *Lepeophtheirus salmonis*, is a marine ectoparasite that infests commercially produced salmonids, subsequently causing high economic losses for the industry [1]. This sea louse has reduced host specificity, and its lifecycle is divided into three planktonic stages (two nauplius phases and an infective copepodid phase) and five parasitic stages (four chalimus phases and an adult phase) [2]. The planktonic stages survive on endogenous reserves, but when the infective copepodid settles onto a host, it molts and extrudes a frontal filament with which it attaches itself to the host [2]. The chalimus stage develops attached to the host and feeds on mucus and skin from the area confined by the frontal filament. In contrast, mature adults are no longer attached to the host and can move freely over the host, thus increasing the area available for feeding. During the parasitic stages, *C*. *rogercresseyi* feed on the mucus, skin, and blood of the host [3], bringing as a consequence irritation, increased mucus secretion, abrasions, the loss of scales, skin disruptions, and wounds for the host, all of which affect the barrier function of the skin and fish osmoregulation [4]. This ultimately facilitates secondary infections, either indirectly or directly, as through a vector [5].

The control of caligidosis, or an infection of *C*. *rogercresseyi*, has primarily been through the use of chemical treatments, one of which is the pyrethroid deltamethrin. Apart from the environmental damage inherent to any large-scale therapeutic drug administration, deltamethrin has also been shown to induce treatment resistance in sea lice [6,7], thus highlighting the urgent need for alternative control strategies. As with any other parasite, the parasitic capacity and infectious success of *C*. *rogercresseyi* depends on its ability to eliminate and/or reduce host defenses so as to attach and remain on the host. The mechanisms employed by *C*. *rogercresseyi* to invade the host and evade subsequent defense responses are yet unclear, although Maldonado et al. [8] reported the presence of serine protease inhibitor-like genes differentially expressed throughout ontogenic development, which could represent a strategy for evading host defenses. Moreover, studies have shown that the saliva of parasitic arthropods is mainly composed of proteases, phosphatases, and prostaglandins [9] that have been shown to immunomodulate the host and facilitate the infestation process [10]. Additionally, pathogenicity has been related to protease activity in a number of different parasitic species [11], such as the ameba *Entamoeba histolytica* [12], the fluke *Fasciola hepatica* [13], the anisakid *Anisakis simplex* [14], and the myxozoan *Myxobolus cerebralis* [15].

Proteases are hydrolytic peptides that catalyze the cleavage of macromolecular unions between proteins and oligomer peptides. Proteases play a direct role in the growth and survival of parasites and are also considered essential during the most critical moments of the parasite-host interaction [16]. Of the different protease families, the cathepsins are among the most studied. These participate in protein degradation in lysosomes or endosomes at a low pH, as well as in cytosol and the nucleus. The cathepsins are divided into several groups according to catalytic mechanisms, and these include the serine (A and G), threonine, aspartate (D and E), and metallo- and cysteine proteases (B, C, H, F, K, L, O, S, V, and W).

In mammals, proteolytic enzymes present a regulatory role in normal biological processes such as protein turnover and cell proliferation, migration, and death [17,18], resulting in their obligate participation in pathological conditions such as cancer [19] and other degenerative diseases such as atherosclerosis [20] and Alzheimer’s [21]. On the other hand, proteases in invertebrates can play a role in tissue penetration and digestion, molting, and in evading a host’s immune response [22–25]. For example, in the protozoan parasite *Trypanosoma brucei*, a cathepsin L participates in the process of blood-brain barrier crossing [26], whereas a cathepsin B regulates iron acquisition [27]. The roles of cathepsins in hematophagous parasites have been described in trematodes such as *Schistosoma mansoni*, where a cathepsin B was linked to hemoglobin degradation [28], and in the hookworm *Necator americanus*, where a cathepsin D was found not only to degrade hemoglobin but also digest connective tissue proteins [29]. In addition to this, invertebrate cathepsins have reported roles in the autophagy processes [30], extracellular digestion [31], vitellogenesis regulation, and the yolk protein process [25]. In the European salmon louse *L*. *salmonis*, cathepsins L [32] and B [33] have been described, and although their roles in parasitic success have yet to be uncovered, the emergent role of cathepsins as potential drug targets [34] stresses the need to investigate the presence and function of cathepsins in the sea louse *C*. *rogercresseyi*, with the goal of achieving an efficient control of this fish ectoparasite.

The present study performed a comprehensive transcriptome survey of cathepsins and their expression patterns in *C*. *rogercresseyi* throughout ontogenic development using high-throughput sequencing.

## Materials and Methods

### Ethics statement

Animal procedures were conducted in accordance with the guidelines and approved by the ethics committee of University of Concepción. Samples from the adult male and female sea lice were collected from a commercial farm located in region X in Chile (41°40’48.5”S; 73°02’31.34”O¨). The permissions for the sea lice collections were authorized by Marine Harvest S.A, Ruta 226, Km. 8, Camino El Tepual, Puerto Montt, Chile. All efforts were made to minimize discomfort and suffering to the animals during handling and manipulation.

### 2.1 Salmon lice culturing and RNA extraction

Female specimens of *C*. *rogercresseyi* were collected from recently harvested fish at a salmon farm. Prior to the collection of salmon lice, fish were anaesthetized. The technical details of transported to the laboratory, egg string collection and culturing were described in a previous study [35]. Herein, twenty individuals from each instars of *C*. *rogercresseyi* including copepodid, chalimus 1–4 and adult were separately collected. Immediately after sampling, each salmon lice stage were pooled into two biological replicates in 1 mL of RNAlater stabilization solution (Ambion, USA) and stored at −80°C. Total RNA was extracted from pools using the Ribopure kit (Ambion, Life Technologies, USA) following the manufacturer's instructions.

### 2.2 Transcriptome sequencing from *C*. *rogercresseyi*

The encoded cathepsin genes were identified from the cDNA library for *C*. *rogercresseyi*. The library was established using Illumina MiSeq sequencing technology from the 6 developmental stages (nauplius I and II, copepodid, chalimus, and adult female and male). The technical details of library construction, including total RNA extraction from a tissue pool, mRNA purification, cDNA synthesis, and sequencing, were described in a previous study [35]. Sequence assembly was carried out using the CLC Genomics Workbench software (Version 7.1, CLC Bio, Denmark). For this, *de novo* assembly was applied with an overlap criterion of 70% and a similarity of 0.9 to exclude paralogous sequence variants (PSVs). Furthermore, the settings used were a mismatch cost of 2, deletion cost of 3, insert cost of 3, minimum contig length of 200 bp, and a trimming quality score of 0.05. After the assembly process, singletons were retained in the data set as possible representatives of low-expression transcript fragments. However, the sequence redundancy of these fragments was removed using the Duplicate Finder application incorporated in the Geneious v5.1.7 software (Biomatters, Auckland, New Zealand). To gauge the number of transcripts and to determine gene function, BLASTx analysis was carried out on consensus sequences. Consensus sequences from the *C*. *rogercresseyi* transcriptome were annotated to the UniprotKB/Swiss-Prot Database (http://uniprot.org) enriched with EST data for crustaceans to determine putative gene descriptions. A cutoff E-value of 1E-05 was used.

### 2.3 Data deposition

The sequences were deposited in the Sequence Read Archive (SRA) (http://www.ncbi.nlm.nih.gov/sra) under the accession number SRR1106551. The *de novo* assembly sequence data are available from corresponding author on request.

### 2.4 Identification of putative cathepsin-like transcripts

To determine *C*. *rogercresseyi* gene homology with other cathepsins previously sequenced in crustacean species, tBLASTn analysis was performed against crustacean EST-datasets downloaded from the NCBI, and specifically against ESTs encoding for putative cathepsin proteins. The tBLASTn results were parsed to retain contigs with an E-value < 1E-10. Additionally, EST mining of crustacean cathepsins was conducted using the online BLAST program and the tBLASTn algorithm (http://www.ncbi.nlm.nih.gov/) by applying known cathepsin sequences as a query. The structural motifs in proteins were analyzed with the SMART online tool (http://smart.embl-heidelberg.de/) [36].

### 2.5 Amino acid sequence and phylogenetic analyses of the *C*. *rogercresseyi* cathepsin family

Protein alignments and phylogenetic tree were constructed using the MUSCLE algorithm tool [37] included in the Geneious v5.1.7 software (Biomatter, New Zealand). One sequence from each of the found cathepsins was chosen to represent the most abundant groups, and these were the *Cr-CatB4*, *Cr-CatZ10*, *Cr-CatD1*, *Cr-CatD6*, *Cr-CatL2*, and *Cr-CatK1* sequences (see S1 Table for further details). These sequences were chosen based on their length, highest Blast score, lowest e-value, and the presence of characteristic, conserved motifs in all of the studied *C*. *rogercresseyi* developmental stages. Exceptionally, two different D-like cathepsin transcripts were chosen, and cathepsin L sequences were used for alignment against arthropod sequences deposited in the public GenBank database (S1A and S1B Fig). The phylogenetic tree was constructed based on a Blosum62 matrix, with a gap open penalty of 12, gap extension penalty of 3, ends-free global alignment, Jukes–Cantor genetic distance model, and the neighbor-joining method. The data were bootstrapped 1000 times to estimate the internal stability of each node.

### 2.6 RNA-seq analysis of cathepsin-like genes

To identify differentially expressed cathepsin-like genes, trimmed reads for each developmental stage (nauplius I and II, copepodid, chalimus and adult female and male) were mapped against cathepsin sequences using CLC Genomic Workbench software (Version 7.1, CLC Bio, Denmark), with a significant annotation (E-value >1E-10) to the selected candidate genes. The RNA-seq was performed with a minimum length fraction of 0.6 and a minimum similarity fraction (long reads) of 0.5. The gene expression was quantified by reads per kilobase of exon model per million mapped reads (RPKM). The proportion-based test method were conducted to identify the differentially expressed genes among nauplius I and II, copepodid, chalimus and adult stages of developmental with false discovery rate (FDR)-corrected P value <0.05. In order to identify differences between stages, RNA-*seq* analyses were performed separately for Nauplius I and II-Copepodid, Copepodid-Chalimus and Female-male. Values obtained in RPKM were normalized and transformed (log2) and fold change between pair of stages was calculated. The Manhattan metric distance was used for hierarchical cluster analysis and genes were classified as differentially expressed if they exhibited more than one fold change between the two samples, and statistical significance at P <0.01 based on the Kal's test [38] between the pairs nauplius and copepodid, copepodid and chalimus and adult female and male.

### 2.7 qPCR Validation

The mRNA relative expression of selected cathepsins transcripts was analyzed to validate the differential expression profiles. Seven contigs of the cathepsins (generated by Illumina transcriptome sequencing) with higher differential expression between developmental stages were selected and measured by quantitative real time PCR. Specific primers were designed with Primer3 tool included in the Geneious Pro v. 5.1.7 software and *β tubulin* gene was used as endogenous control (Table 1). The qPCR runs were performed with StepOnePlus (Applied Biosystems, Life Technologies, USA) using the comparative 2ΔCt method [39]. To perform this, we conducted five cDNA dilution series, beginning with 80 ng of template and dilution factor of 1:5. Each reaction was conducted with a volume of 10 μL using the Maxima SYBR Green/ROX qPCR Master Mix (Thermo Scientific, USA). The amplification conditions were as follows: 95°C for 10 min, 40 cycles at 95°C for 30 s, 60°C for 30 s, and 72°C for 30 s. Significant differences between relative expression along development stages were analyzed by test of means Tukey HSD with the JMP software v.9.0 (SAS Institute Inc., USA) with P <0.05.

**Table 1:** Forward and reverse qPCR primer sequences used for expression analysis from different life stages of *Caligus rogercresseyi* cathepsins.

Primers	Sequence (5’-3’)
***Cr-CatB1-F***	CTAATAGTATCCTGTCATCG
***Cr-CatB1-R***	AGGTAGTTAGAAGAAGTCTC
***Cr-CatD1-F***	CTCTTCCATCTTCCTTATAG
***Cr-CatD1-R***	CAGAAGTTTGAGGTCATC
***Cr-CatD2-F***	GAGATCCATTCCCATAAATC
***Cr-CatD2-R***	CAACATTGACTTCACTCTG
***Cr-CatZ10-F***	CCAATCTAGTCATCAACTC
***Cr-CatZ10-R***	CTCTTAACACATGACTCTC
***Cr-CatL1-F***	GATCTACACTCTTTCCCTA
***Cr-CatL1-R***	CTCCTTAATACCAACTACC
***Cr-Catl26-F***	CAGTGAATAATAGTACCCG
***Cr-Catl26-R***	GGCCAATACTTTATGAGAG
***Cr-Catl27-F***	GGAGATCAAGGATACATCAA
***Cr-Catl27-R***	TCTCCAGTATCTGTAATTGG
***B-tubulinF***	ACCGCACATGGTGAGTGATA
***B-tubulinR***	CTCGTAGAAAACAGGGACGA

### 2.8 Single nucleotide polymorphism (SNP) mining from cathepsin-like genes

SNP candidates were identified from the *de novo* assembly of the six developmental stages of *C*. *rogercresseyi* using the CLC Genomics Workbench software (Version 7.1, CLC Bio, Denmark) and quality base variant detection. SNPs mining followed a neighborhood radius of 11, a maximum gap and mismatch count of 2, a minimum neighborhood quality of 15, a minimum coverage of 20, a minimum variant frequency (%) of 25.0, and a maximum expected alleles (ploidy) of 2. The frequencies of SNPs found in all of the contigs were calculated using the same software and were tabulated in Excel spreadsheets for analysis.

## Results

### 3.1 Identification of cathepsin sequences from *C*. *rogercresseyi*

Fifty-six putative, partial cathepsin-like sequences were found in the *C*. *rogercresseyi* transcriptome. For use in future discussions, the cathepsin-like sequences discovered in this study were denoted *Cr-CatL*, *Cr-CatB*, *Cr-CatD*, *Cr-CatK*, *Cr-CatZ*, *Cr-CatW*, and *Cr-CatS* (S1A Table and S2B Table). Structural analysis through the SMART program showed that the group of *Cr-CatL*, *Cr-CatB*, *Cr-CatK*, *Cr-CatZ*, and *Cr-CatS* sequences had the Papain family cysteine protease (Pept_C1). The *Cr-CatF*, *Cr-CatK*, and *Cr-CatL* group additionally presented the inhibition motif (Inhibitor I29) (S1A Table), while the majority of the *Cr-CatD* group showed a retropepsin-like domain.

### 3.2 *In silico* evidence for other crustacean cathepsins

**Fig 1:**
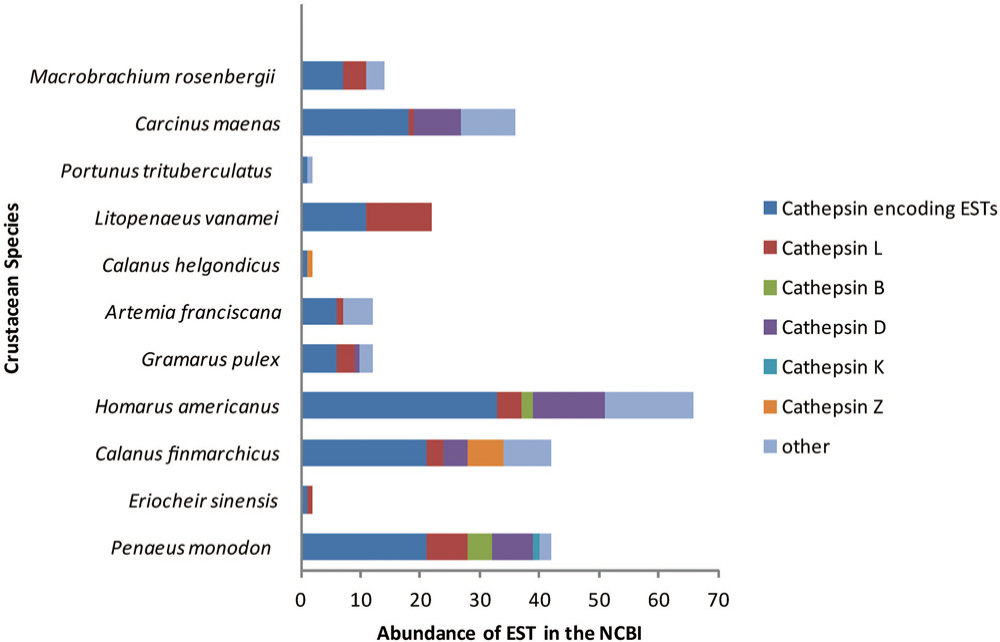
Diversity and abundance of cathepsin encoding transcripts in crustaceans based on available expressed sequence tag in NCBI (dataset with >10,000 ESTs as of 10^th^ June 2014).

**Fig 2:**
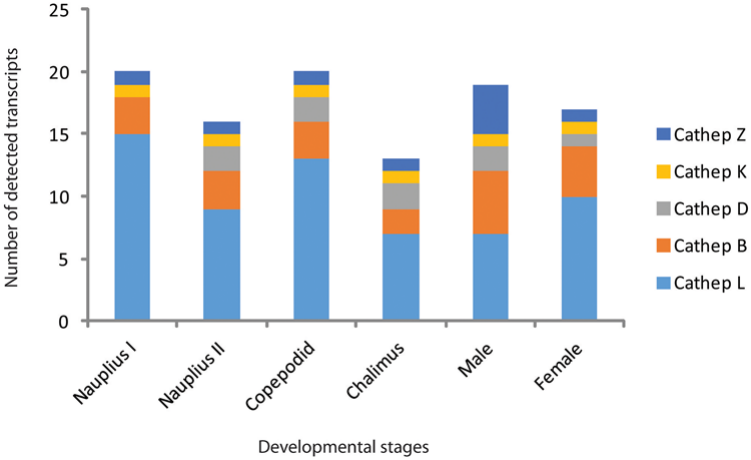
Distribution and abundance of cathepsin-like transcript identified in *Caligus rogercresseyi* in the six developmental stages.

The 56 cathepsins sequences identified for *C*. *rogercresseyi* were used as a reference for investigating their presence in other crustacean species. Of the 18 species present in the crustacean EST database, only 11 have reported EST sequences for cathepsins (Fig 1). The cathepsin L group was the most abundant between analyzed species, whereas cathepsin B was found in only *Penaeus monodon* and *Homarus americanus*, while cathepsin K was found only in *Penaeus monodon* (Fig 1). Similar to that described in other crustaceans, L-like cathepsin transcripts in *C*. *rogercresseyi* were the most abundant across all eight developmental stages, followed by cathepsins B and Z (Fig 2).

### 3.3 Phylogenetic and amino acid sequence analyses

**Fig 3:**
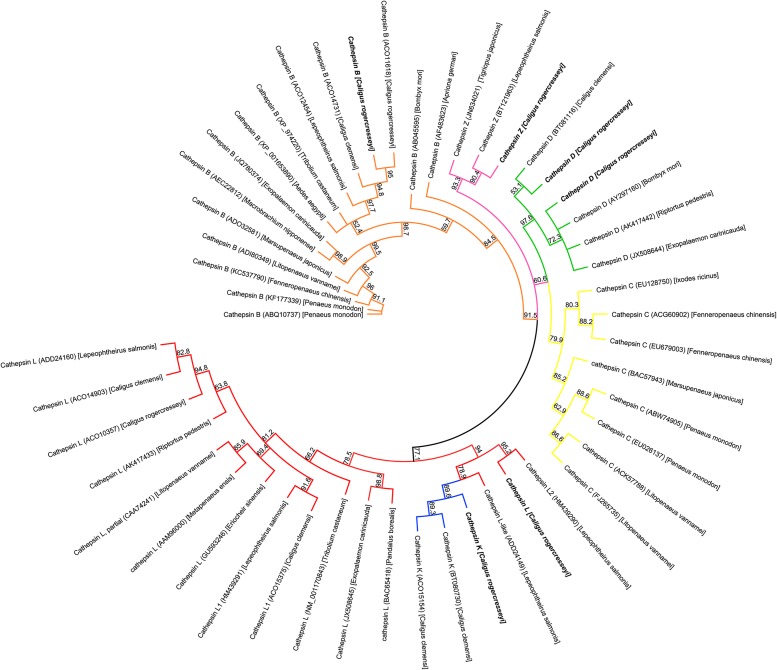
Phylogenetic relationships between *Caligus rogercresseyi* cathepsins-like sequences and the cathepsins sequences of other crustacean species. Clades for each cathepsin are differentiated by colour as follow: cathepsin B—orange; cathepsin C—yellow; cathepsin D—green; cathepsin K—blue; cathepsin Z—pink; and cathepsin L—red. The tree was constructed by Neighbour-joining method and confirmed by Bootstrap with 1000 iteractions. Support values at the nodes are shown and *C*. *rogercresseyi* cathepsins transcripts used were *Cr-CatB4*, *Cr-CatZ10*, *Cr-CatD6*, *Cr-CatD1*, *Cr-CatL2* and *Cr-CatK1* (see S1 Table for detailed information on each transcript).

The phylogenetic analysis investigated the evolutionary relationships of the cathepsin-like sequences found in *C*. *rogercresseyi*, and the robustness of the nodes was supported by high bootstrap values (Fig 3). Each cathepsin group was differentiated by colors, and in the clade dominated by cathepsin L (red color), there was an evident sub-clade that included cathepsin K (blue color) from *C*. *clemensi*. Additionally, cathepsin Z formed part of a remote clade of the group that also included cathepsin D and C, while the amino acid sequences of cathepsin B were found distant to other intracellular hydrolase clades (Fig 3). Moreover, the alignment of cathepsin L sequences of arthropod organisms showed that the *C*. *rogercresseyi* transcript for *Cr-CatL2* presented high levels of conservation. The catalytic triad of cysteine, histidine, and asparagine was highly conserved in all analyzed species as the active site cysteine is incorporated in the highly conserved peptide sequence CGSCWAFS as the signal sequence GCNGG (S1A Fig), and, following an analysis of the aligned sequence in the SIGNALP server (http://www.cbs.dtu.dk/services/SignalP/), the cleavage site of the signal peptide was predicted between positions 18/19.

### 3.4 Differential gene expression among developmental stages of *C*. *rogercresseyi*

**Fig 4:**
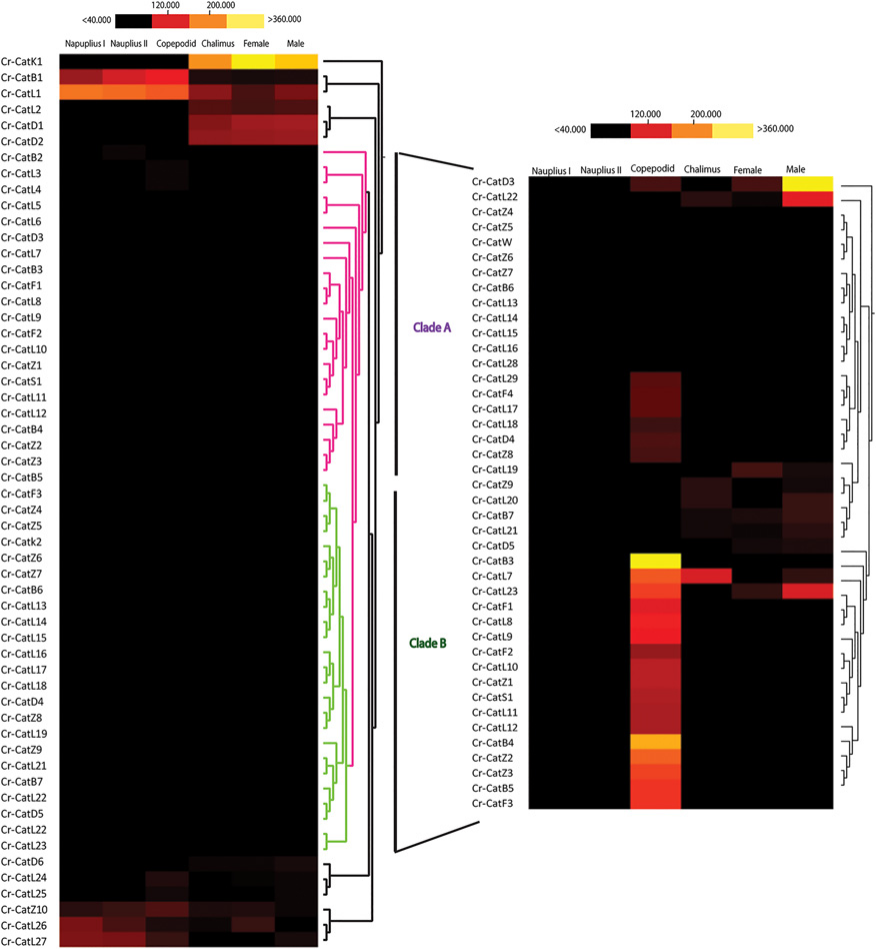
Heat maps of cathepsin-like transcripts from the six developmental stages of *Caligus rogercresseyi* representing all cathepsins found (A), and a magnification of the cathepsins found in cluster A and B (B). Transcript abundance is represented as RPKM values and color scales show relative transcript expression.

**Fig 5:**
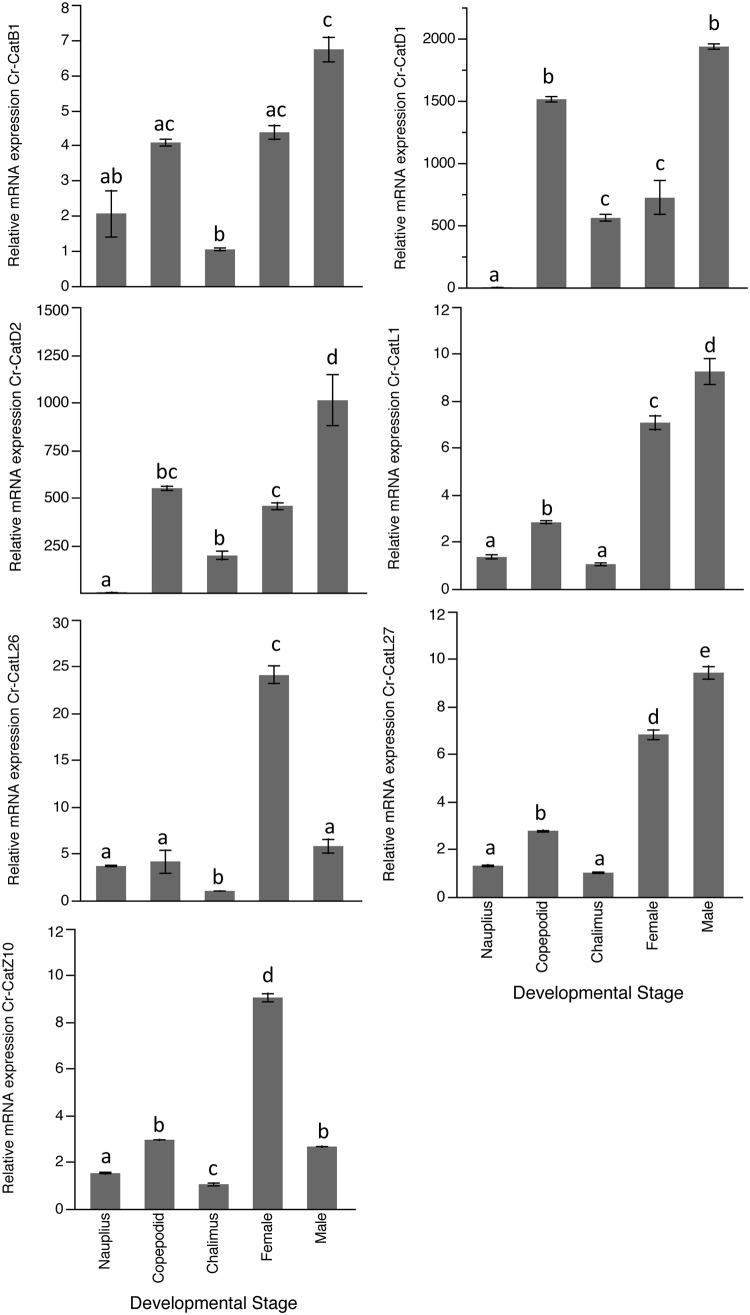
Relative expression of *Caligus rogercresseyi* selected cathepsins mRNA along developmental stages. Data are presented as mean ± SEM and different letters indicate significant differences in the relative expression of the cathepsins between life stages (Tukey HSD, P< 0.05).

The 56 cathepsin-related transcripts found in *C*. *rogercresseyi* were used for RNA-*seq* analysis, including cathepsin-like transcripts from groups L, D, F, B, Z, H, K, and S (see S1A Table for transcript details). Analysis revealed that the transcripts were differentially expressed between six developmental stages of *C*. *rogercresseyi* (Fig 4A). A sub-experiment with the 42 sequences included in clusters A and B revealed underlying differential expression patterns mainly associated with the copepodid stage, where several cathepsin B, D, F, K, L, S, and Z-like genes were upregulated (Fig 4B). A total of 26 cathepsin-like genes were differentially expressed throughout the different stages. Between the nauplius and copepodid stages, five genes were found to be up-regulated while another five were down-regulated (Table 2), with *Cr-CatL2* being highly downregulated. On the other hand, between the copepodid and chalimus stages, five genes were upregulated while 13 were downregulated (Table 3). Of these, *Cr-CatL2* was the most significantly expressed with an up-regulation of 198-fold. The analysis between adult females and males evidenced that 19 genes were upregulated while five cathepsin-like sequences were downregulated (Table 4), and among these, *Cr-CatL26* was the most downregulated by more than 60-fold. The majority of the genes that were significantly regulated were those associated with the adult stage, while for the nauplius and copepodid stages, only ten cathepsin-like transcripts were differentially expressed. Finally, the seven cathepsin-like selected genes mRNA expression (Fig 5) validated the *in silico* differential expression observed in the study.

**Table 2:** Genes showing differential transcription expression between nauplius I- II and copepodid stages of *Caligus rogercresseyi*.

	RPKM		
ID	Nauplius I-II	Copepodid	FC
***Up- regulated***			
*Cr-CatD6*	2102,32	6940,23	2,96
*Cr-CatD1*	2654,55	7010,59	2,37
*Cr-CatK1*	2139,41	2825,07	1,19
*Cr-CatZ10*	48970,52	59740,73	1,09
*Cr-CatB1*	145744,27	162668,93	1,01
***Down-regulated***			
*Cr-CatL2*	2630,31	289,44	-10,13
*Cr-CatL26*	58891,95	23491,82	-2,79
*Cr-CatL27*	87896,63	49397,28	-1,98
*Cr-CatB2*	13880,41	9164,45	-1,69
*Cr-CatL1*	241570,61	229736,15	-1,17

**Table 3:** Genes showing differential transcription expression between copepodid and chalimus stages of *Caligus rogercresseyi*.

	RPKM		
ID	Copepodid	Chalimus	FC
**Up- regulated**			
*Cr-CatL2*	289,44	63944,18	198,86
*Cr-CatK1*	2825,07	270820,95	86,29
*Cr-CatD2*	5013,69	100833,39	18,10
*Cr-CatD1*	7010,59	95712,65	12,29
*Cr-CatD6*	6940,23	14520,44	1,88
***Down-regulated***			
*Cr-CatL27*	49397,28	734,97	-74,67
*Cr-CatL3*	13988,95	2480,33	-6,27
*Cr-CatB1*	162668,93	29519,26	-6,12
*Cr-CatL6*	7073,28	1350,61	-5,82
*Cr-CatB6*	9164,45	2166,55	-4,70
*Cr-CatL24*	29299,08	7392,75	-4,40
*Cr-CatZ10*	59740,73	25434,95	-2,61
*Cr-CatL1*	229736,15	97996,43	-2,60
*Cr-CatL5*	7683,32	3353,36	-2,54
*Cr-CatL4*	13499,17	6243,88	-2,40
*Cr-CatL26*	23491,82	16660,99	-1,57
*Cr-CatL7*	1970,59	1397,59	-1,57

**Table 4:** Genes showing differential transcription expression between adult female and male stages of *Caligus rogercresseyi*.

	RPKM		
ID	Female	Male	FC
***Up-regulated***			
*Cr-CatL22*	78,11	1633,03	21,20
*Cr-CatB2*	226,79	3017,44	13,49
*Cr-CatD3*	558,78	7168,91	13,01
*Cr-CatL23*	355,17	1518,88	4,34
*Cr-CatL21*	78,11	296,91	3,85
*Cr-CatL27*	7898,85	23007,22	2,95
*Cr-CatL3*	1557,84	4441,43	2,89
*Cr-CatB7*	185,00	439,54	2,41
*Cr-CatD5*	99,99	190,06	1,93
*Cr-CatL5*	702,06	1167,59	1,69
*Cr-CatL1*	57451,56	89399,85	1,58
*Cr-CatL4*	5228,86	7122,51	1,38
*Cr-CatD6*	16591,12	19913,04	1,22
*Cr-CatL24*	10541,37	12721,13	1,22
*Cr-CatL2*	53807,08	60202,09	1,13
*Cr-CatB1*	22679,38	23923,96	1,07
*Cr-CatD2*	104435,33	108749,48	1,06
*Cr-CatD1*	110861,39	113847,14	1,04
***Down-regulated***			
*Cr-CatL26*	47751,32	742,99	-63,39
*Cr-CatL19*	535,28	169,56	-3,11
*Cr-CatZ10*	28363,43	13556,11	-2,06
*Cr-CatK1*	342640,13	303499,92	-1,11
*Cr-CatL6*	969,47	921,33	-1,04

### 3.5 SNPs identification

A total of 140 SNP type variants were identified in transcripts annotated for cathepsins distributed throughout six developmental stages of *C*. *rogercresseyi* (Table 4). Putative SNPs were classified in variants of transitions (A/G or C/T) since the proportions of A/C, G/T, and C/G transversions were similar in the six investigated developmental stages. Putative SNPs in cathepsin L, B, Z, and K transcripts were found in the nauplius I—II stages, while in the copepodid stage, SNPs were also identified in cathepsin D. In the chalimus, SNPs were found in cathepsins L, D, and Z. In adult males variants were identified in cathepsins L, K, and Z, and in females these were additionally found in cathepsin B. From the 140 identified SNPs, 43% were non-synonymous variations, while synonymous changes represented 13% and were distributed among the six examined life stages of the sea louse (Table 5).

**Table 5:** SNPs identification cathepsins- like sequences from *Caligus rogercresseyi*

ID	Consensus	Consensus	Frecuency	Substitution
***Nauplius I***				
*Cr-CatL1*	340	A/G	26,97	Synonym
	1144	A/G	37,36	Synonym
	1158	C	54,92	3´UTR
	1161	G	54,24	3´UTR
	1162	A/G	49,06	3´UTR
	1174	T/G	52,08	3´UTR
	1183	G/T	54,35	3´UTR
*Cr-CatB1*	1137	T/C	29,44	5´UTR
*Cr-CatZ10*	940	G/T	33,33	5´UTR
	1076	G/T	37,21	5´UTR
	1876	C/T	36,17	5´UTR
	2462	C/A	40,00	5´UTR
	2775	A/G	41,94	5´UTR
	3154	A/G	39,13	5´UTR
	3370	A/G	34,62	5´UTR
	3457	A/T	25,71	5´UTR
	4739	C/A	35,29	No synonym
	4864	C/A	43,75	Synonym
	5020	G/A	39,34	Synonym
	5158	T/A	44,44	Synonym
	5293	T/A	35,71	Synonym
	5311	T/G	44,64	Synonym
	5385	A/G	26,67	No synonym
	5558	T/C	46,58	Synonym
	5777	C/G	43,52	No synonym
	5778	A/G	43,52	No synonym
	6574	A/T	32,31	Synonym
*Cr-CatL13*	870	G/T	25,53	No synonym
	1048	A/G	45,65	No synonym
	1176	T/A	30,43	No synonym
***Nauplius II***				
*Cr-CatL1*	57	CT	51,35	3´UTR
	65	T/C	50,00	3´UTR
	67	T/G	50,00	3´UTR
	86	C/A	37,10	3´UTR
	95	A/C	37,50	3´UTR
	107	C/T	30,77	3´UTR
	125	T/C	27,74	3´UTR
	929	T/C	30,59	No synonym
*Cr-Cat Z10*	22	TA	31,34	Deletion
	104	T/A	45,13	No synonym
	900	T/C	32,54	Synonym
	901	G/C	32,54	No synonym
	1120	A/G	44,55	No synonym
	1232	G/A	25,81	No synonym
	1367	A/C	44,44	No synonym
	1385	A/T	42,11	No synonym
	1520	T/A	49,07	No synonym
	1658	C/T	46,23	No synonym
	1814	G/T	34,65	No synonym
	1939	T/G	45,76	No synonym
*Cr-CatL13*	889	G/T	28,57	No synonym
	1016	A/G	35,29	No synonym
*Cr-CatL26*	445	T/A	33,33	No synonym
	926	G/A	37,23	No synonym
***Copepodid***				
*Cr-Cat L1*	341	A/G	34,76	No synonym
	1159	C	49,38	Insertion
	1203	A/C	27,59	Synonym
*Cr-CatL4*	137	A/G	32,20	No synonym
*Cr-CatL26*	264	C/T	36,36	5´UTR
	1267	A/C	40,00	5´UTR
	1364	T/G	28,57	5´UTR
	1365	G/A	28,57	5´UTR
	1424	T/A	37,50	5´UTR
	1946	C/T	33,33	5´UTR
	2063	T/A	36,36	5´UTR
	2098	C/G	33,33	5´UTR
	2985	T/A	33,87	Synonym
	3202	C/T	37,74	No synonym
	3466	G/A	31,88	No synonym
*Cr-CatB2*	267	T/C	34,33	Synonym
*Cr-CatD6*	131	T/C	37,50	No synonym
	461	A/G	45,83	No synonym
	509	T/C	42,31	No synonym
	651	A/T	40,00	No synonym
*Cr-CatL13*	245	C/T	48,47	No synonym
	423	C/A	25,47	Synonym
*Cr-CatZ10*	669	C/T	45,00	3´UTR
	1648	T/A	47,90	3´UTR
	2444	T/C	44,41	3´UTR
	2445	G/C	44,41	3´UTR
	2664	A/G	39,93	3´UTR
	2911	A/C	43,59	3´UTR
	2929	A/T	41,54	3´UTR
	3064	T/A	45,61	3´UTR
	3202	C/T	38,37	3´UTR
	3358	T/G	50,00	3´UTR
	3483	T/G	46,72	3´UTR
***Chalimus***				
*Cr-CatL1*	130	C/T	43,18	3´UTR
	289	C/T	34,39	No synonym
	319	T/A	25,93	No synonym
*Cr-Cat Z10*	1144	T	27,27	Insertion
	1229	T/A	39,27	3´UTR
	1301	A/G	31,08	3´UTR
	2025	T/C	36,08	Synonym
	2026	G/C	36,08	No synonym
	2357	G/A	30,40	No synonym
	2492	A/C	44,78	No synonym
	2510	A/T	31,53	No synonym
	2645	T/A	47,51	No synonym
	2720	G/T	27,60	No synonym
	2783	T/C	35,12	No synonym
*Cr-CatL25*	505	C/T	37,50	No synonym
	973	C/T	32,14	No synonym
	1024	T/A	27,59	3´UTR
	1032	G/A	27,59	3´UTR
*C-CatL13*	409	G/T	40,59	No synonym
	1425	G/A	38,78	Synonym
*Cr-CatD6*	672	A/T	26,09	No synonym
	1306	T/C	42,11	No synonym
	1339	T/C	26,85	No synonym
	1561	G/A	42,37	No synonym
	1728	G/A	41,25	Synonym
*Cr-CatB2*	1262	C/T	49,49	No synonym
***Male***				
*Cr-CatK1*	616	G/A	29,95	No synonym
	1352	C/T	28,55	3´UTR
*Cr-CatL25*	536	G/A	27,50	3´UTR
*Cr-CatZ10*	77	A/C	38,95	5´UTR
	202	C/A	27,88	5´UTR
	358	G/A	46,60	5´UTR
	496	T/A	34,55	Synonym
	649	T/G	49,79	3´UTR
	784	C/T	41,67	3´UTR
	896	C/T	43,51	3´UTR
***Female***				
*Cr-Cat Z10*	203	C/A	44,55	No synonym
	328	C/A	45,72	No synonym
	484	G/A	49,32	No synonym
	622	T/A	47,99	No synonym
	757	T/A	39,81	No synonym
	910	C/T	26,59	No synonym
	1022	C/T	38,20	No synonym
	1241	C/G	38,19	No synonym
	1242	A/G	38,19	No synonym
	2038	T/A	45,59	No synonym
	2234	T/A	50,00	3´UTR
*Cr-CatK1*	128	T/C	34,83	5´UTR
	1094	A/C	26,42	3´UTR
*Cr-CatL14*	452	C/T	50,00	3´UTR

## Discussion

The ability to overcome and modulate a host's protective defenses is the key to the infectious success of parasites. Proteases are deployed in essential tasks such as tissue penetration and nutrient digestion, as well as in evading the immune response of the host [22–25]. This study reported for the first time the presence and differential expression of a group of proteases, the cathepsins, in *C*. *rogercresseyi*. Fifty-six cathepsin-like sequences were identified and distributed in the cysteine protease groups B, F, L, Z, and S and in the aspartic protease group D.

Cysteine proteases present a signal sequence that allows for the orientation of the pro-peptide towards different storage sites. The present results, obtained from tBLASTx and SMART analyses, are congruent with this since the presence of the I29 inhibitor sequence was found in relation to a pro-peptide in inactive cathepsins, but this could activate through the proteolytic cleavage of the N-terminal pro-peptide [40]. This is in agreement with other cysteine proteases, such as that found in *L*. *salmonis* which presents two cathepsin L sequences with a pro-peptide sequence in the extreme N-terminal [32]. In addition to this, SMART analysis of identified aspartic proteases revealed a retropepsin-like domain, which is characteristic of aspartic proteases [41]. Members of the cathepsin L group have been described as some of the most common proteases among arthropods [42]. Indeed the majority of cathepsin ESTs available in the NCBI belongs to cathepsins L and B. The sea louse *C*. *rogercresseyi* was no exception, and in the present study, L cathepsins were the most abundant across all of the analyzed life stages of this copepod. This find was based on a multiple alignment of the *Cr-CatL2* sequence with sequences from other arthropods, and the sequence obtained presented conserved domains characteristic of L cathepsins, including the signal peptides MKALSVLACVVA, GCNGG, and CGSCWAFS. The GCNGG sequence had a cysteine residue implied through the formation of a disulfur bridge, therefore indicating a structural function. Meanwhile, the CGSCWAFS sequence corresponded to the active cysteine site and the catalytic triad CHN. However, it was not possible to identify the ERFNIN motif, which is linked to auto-inhibitory functions, or the GNFD motif, which regulates the processing and folding of C1A proteases [43], even though these were found in the sea louse *L*. *salmonis* in sequences *LsCL1* and *LsCL2* [32].

The phylogenetic analysis of the transcripts annotated for cathepsins in *C*. *rogercresseyi* showed an evident relationship with the amino acid sequences of cathepsins reported for other arthropods. In the cathepsin L clade, another sub-clade was formed based on cathepsin K, and this is in line with the close relationship reported between cathepsins K and S with cathepsins L through the shared auto-inhibitory motif ERFNIN [11]. On the other hand, the other *C*. *rogercresseyi* identified cathepsin sequences (D, Z, and B) formed independent clades with the amino acid sequences reported for each arthropod species used in this study.

Cysteine proteases have been identified as potential targets for drug or vaccine development against several parasites [44] as their function appears to be essential in a variety of important biological processes within the parasite, such as in molting, cuticle remodeling, embryogenesis, feeding, and immune evasion [45]. The expression analysis among the different life stages showed high *Cr-CatK1* expression in adult females. This is in agreement with other studies in parasites that focused on the relationship between cysteine proteases and embryonic development [46,47]. For instance, in *Caenorhabditis elegans*, the cathepsin *Ce-CPL-1* plays an essential role in yolk protein processing during embryonic development, and a loss of activity leads to enlarged cytoplasm yolk vesicles and embryonic mortality [46]. In addition to this, a dramatic reduction in the production and hatching of eggs occurs when parasites such as *Fasciola hepatica* infected a cathepsin L immunized host [47]. Similarly, the parasite *Brugia malayi* presented a cysteine protease, *Bm_clp*, in the egg yolk during its embryonic development [44]. In turn, *Cr-CatL1*, *Cr-CatL26*, and *Cr-CatL27* cathepsin transcripts from group L, together with *Cr-CatB1*a from cathepsin group B, were found upregulated in the nauplius stages before becoming progressively downregulated throughout development. That adults presented the highest number of differentially expressed genes and that most were up-regulated in males might be due to the higher mobility in male sea lice and the capacity to change hosts [48]. This mobility is associated with defense mechanisms and reproductive success [48] and might translate into the increased expression of genes related to parasitic capacity, such as cathepsins.

Phylogenetic analysis showed cathepsin B sequence similarities between *C*. *rogercresseyi* and other infectious arthropods such as *Aedes aegypti*, *C*. *clemensi*, and *L*. *salmonis*, thus suggesting similarities in functional aspects as well. Cathepsin B has been implicated in the digestion processes of *L*. *salmonis* and in other parasites [33,49], but it has also been associated with embryogenesis, tissue invasion, iron acquisition, immune response evasion, and the molting processes [27,50–53]. In the nematode *Hysterothylacium aduncum*, cathepsin B functions are associated with molting processes due to the variations presented during ontogenic development [45]. Similarly, the high expression found for *Cr-CatL1*, *Cr-CatL26*, *Cr-CatL27*, *Cr-CatB1*, and *Cr-CatZ10* suggests possible involvement in the molting processes or shell remodeling in *C*. *rogercresseyi*, which would evidence the diversity of possible biological functions attributed to cathepsins [25]. Along this same line, the present results showed that a variety of cathepsins from groups Z, L, D, B, K, and S had high expression in the copepodid stage and might therefore be implicated in processes related to evading the host's immune system, feeding, or molting. Indeed, the participation of cathepsin B in the penetration of and migration to new host tissues after detecting juvenile secretions has been reported in the parasite *Fasciola hepatica* [24]. Finally, although cysteine proteases F and S were identified in this study, the functional properties of these in arthropods are poorly studied. In parasites such as *Teladorsagia circumcincta* and Clonorchis sinensis, cathepsin F is one of the most secreted products [54,55], suggesting a participation in host-parasite interactions. The higher differential expression found in the copepodid stage of *C*. *rogercresseyi* would indicate that these cathepsins participate in the parasitic approach towards the host.

Aspartic proteases have been associated with key processes in the lifecycle of various endo- and ectoparasites such as ticks and flatworms [56,57]. However, these proteases have mainly been linked to endocytosis and intracellular protein degradation [58], with participation in processes such as metamorphosis in the silkworm *Bombix mori* [22] or in yolk degradation in the tick *Boophilus microplus* [56,59]. Additionally, aspartic proteases have been related to blood digestion in parasitic mites and ticks [60]. In *Haemaphylasis longicornis*, for example, the aspartic *longepsin* was found to be more expressed in the saliva glands than in the ovary, indicating an antigenic role during hematophagous feeding [56]. Similarly, in some pathogens such as plasmodium [61], *Ancylostoma caninum* [62], and *Schistosoma* [63], it has been demonstrated that these proteases play a key role in hemoglobin proteolysis. RNA-*seq* results obtained for *C*. *rogercresseyi* showed that two of the aspartic proteases from group D, *Cr-CatD1* and *Cr-CatD2*, had higher transcriptomic expression in the chalimus and adult stages, while in the larval stages expression was not elevated. The high expression of these proteases in the parasitic stages suggests involvement in the hemoglobin proteolytic process during the adult feeding process of the parasite, which is supported by evidence of hematophagous behavior in caligid copepods [3].

Given that SNPs might be bound to differences in target genotypes or phenotypes, these are now widely studied [64]. Moreover, these markers have the advantage of being identifiable in non-model organisms through using high-throughput sequencing [65]. In *C*. *rogercresseyi*, 140 SNPs were identified in transcripts annotated for cysteine and aspartic proteases located in the 5'UTR, 3'UTR, or the coding region. From all of the identified variations, 22 SNPs were located in the 5'UTR, 37 in the 3'UTR of the mRNA of the respective cathepsin, and 78 in the coding region of the cathepsin, of which 60 were non-synonymous variations and 18 were synonymous variations. When analyzing the developmental stages of *C*. *rogercresseyi* that presented the highest proportion of SNPs, the copepodid evidenced the most polymorphisms in cathepsin L, whereas nauplius I was the ontogenic stage with the fewest SNPs. Only *Cr-CatL26* in the copepodid stage showed 11 mutations, most of which were in the 5'UTR region. Studies regarding SNPs in the cathepsins of arthropods show a relationship between genotypic variation and the phenotypic response. As an example, *Fenneropenaeus chinensis* presents a synonymous mutation in the ORF of a cathepsin B corresponding to a C/T transition associated with resistance/susceptibility to the white spot virus that affects cultured crustaceans [66]. Moreover, *Cr-CatZ10* was differentially expressed during the developmental stages and interestingly SNPs annotated in the nauplius I stage were the most abundant mutations in this sequence. Contrary to this, the copepodid stage only presented polymorphic variations in the 3'UTR region. In this context, the analysis of the differential expressions between the nauplius I-II and copepodid stages showed a positive regulation in the copepodid stage, while the transcriptomic expression between the copepodid and chalimus showed a downregulation with the development of the copepod to the parasitic stage. Moreover, *Cr-CatZ10* was found more expressed in female adults. These results suggest that the presence of polymorphisms in the 3'UTR region of *Cr-CatZ10* might have effects on the transcriptomic expression of these proteases during the *C*. *rogercresseyi* lifecycle. Indeed, polymorphisms present in the non-coding regions might affect gene transcription through the modification of regions that regulate mRNA stabilization [67].

## Conclusion

The results presented in this study identify the presence of several cathepsin-like sequences in *C*. *rogercresseyi* that are differentially expressed during their ontogenic development stages. The putative roles of the identified cathepsins ranged from host tissue invasion and digestion to evading the host's immune system and to reproductive capacity. In this context, the functional analysis of cathepsins might be a major aid towards selecting target molecules for the development of new drugs or vaccines against *C*. *rogercresseyi* infestations. Nevertheless, further research should be undertaken so as to understand the expression of these proteins in response to host nutrition and immunocompetence, and also to develop a standardized procedure for the unbiased evaluation and manipulation of *C*. *rogercresseyi* digestive proteins.

## Supporting Information

S1 Fig **A**. Amino acid alignment of *Caligus rogercresseyi* cathepsin L-like *Cr-CatL2* against cathepsin L amino acid sequences from several species. **B**. Nucleotide sequence of Cr-Cath L2 from *C*. *rogercresseyi*. The primer used to amplify the sequence are marked with an arrow in forward and reverse sense.
(DOCX)

S1 Table **A**. tBLASTx analysis of *Caligus rogercresseyi* cathepsin-like transcripts. **B**. Sequences of primers used in this study for Cr-Cath L2 sequence.
(DOCX)
