# Supplementary material for: Cathepsin Gene Family Reveals Transcriptome Patterns Related to the Infective Stages of the Salmon Louse Caligus rogercresseyi
Source: PLoS One. 2015 Apr 29;10(4):e0123954. doi: 10.1371/journal.pone.0123954 (PMC4414500; doi:10.1371/journal.pone.0123954)
Supplement: S1 Fig — A. Amino acid alignment of Caligus rogercresseyi cathepsin L-like Cr-CatL2 against cathepsin L amino acid sequences from several species. B. Nucleotide sequence of Cr-Cath L2 from C. rogercresseyi. The primer used to amplify the sequence are marked with an arrow in forward and reverse sense. (DOCX) [file pone.0123954.s001.docx]

**Figure S1A**. Amino acid alignment of *Caligus rogercresseyi* cathepsin L-like *Cr-CatL2* against cathepsin L amino acid sequences from several species.
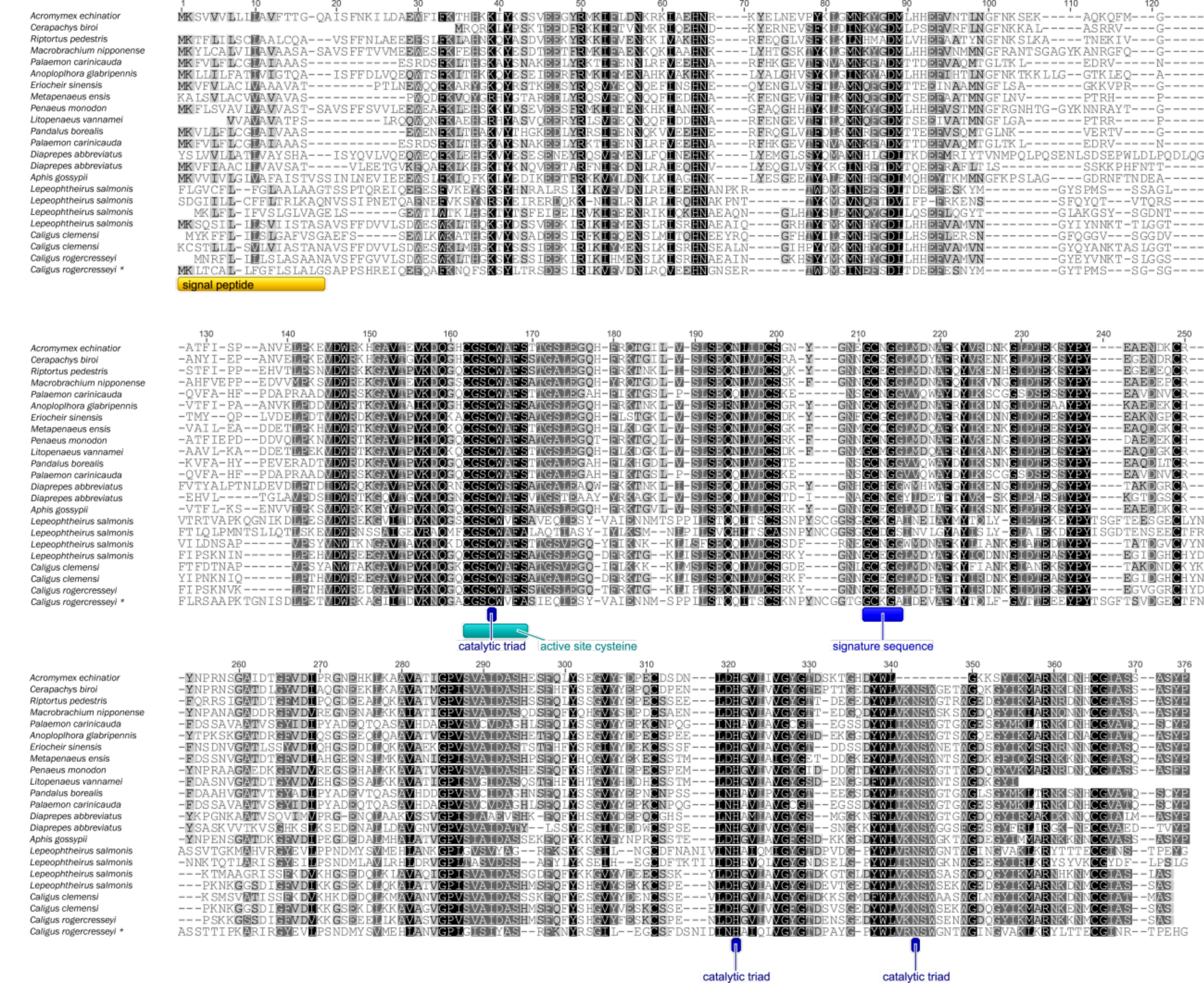


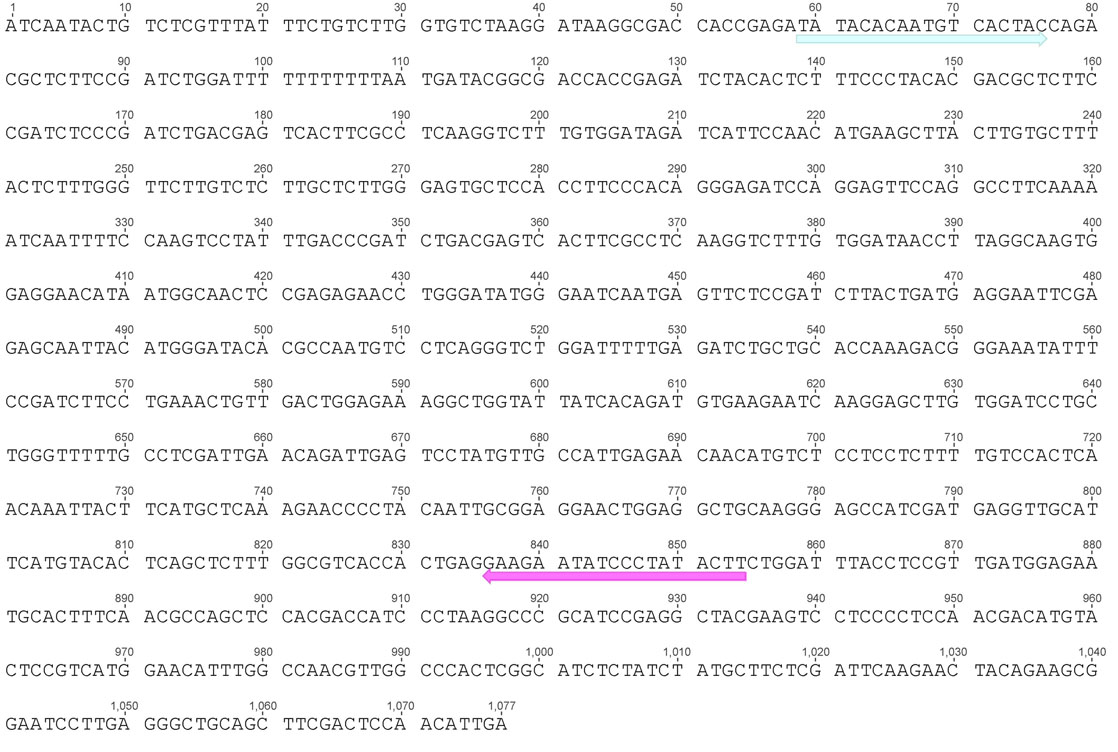
**Figure S2B**. Nucleotide sequence of Cr-Cath L2 from *C. rogercresseyi*. The primer used to amplify the sequence are marked with an arrow in forward and reverse sense.
