# Supplementary material for: Cathepsin Gene Family Reveals Transcriptome Patterns Related to the Infective Stages of the Salmon Louse Caligus rogercresseyi
Source: PLoS One. 2015 Apr 29;10(4):e0123954. doi: 10.1371/journal.pone.0123954 (PMC4414500; doi:10.1371/journal.pone.0123954)
Supplement: S1 Table — A. tBLASTx analysis of Caligus rogercresseyi cathepsin-like transcripts. B. Sequences of primers used in this study for Cr-Cath L2 sequence. (DOCX) [file pone.0123954.s002.docx]

| Table S1A. tBLASTx analysis of *Caligus rogercresseyi* cathepsin-like transcripts. | | | | | | |
| --- | --- | --- | --- | --- | --- | --- |
| Query sequence | Accession no. | Species | Protein description | Blast score | E-value | SMART Analysis |
| Cathepsin-family B | | | | | | |
| Cr-CatB1 | ACO11618 | *Caligus rogercresseyi* | Cathepsin B precursor | 80% | 3e-71 | Propeptide_C1 Superfamily/ Peptidase_C1 |
| Cr-CatB2 | ACO11618 | *Caligus rogercresseyi]* | Cathepsin B precursor | 99% | 7e-46 | Propeptide_C1 Superfamily/ |
| Cr-CatB3 | AHW50664 | *Naegleria fowleri* | CathepsinB-like protein | 54% | 2e-58 | Peptidase_C1 superfamily |
| Cr-CatB4 | EJY81064 | *Oxytricha trifallax* | Cathepsin B | 52% | 1e-38 | Peptidase_C1 superfamily |
| Cr-CatB5 | AHW50664 | *Naegleria fowleri* | Cathepsin B-like protein | 58% | 6e-20 | Peptidase_C1 superfamily |
| Cr-CatB6 | [ACN10219](http://www.ncbi.nlm.nih.gov/protein/223646922?report=genbank&log$=prottop&blast_rank=2&RID=V5RZ92ZG01R) | *Salmo salar* | Cathepsin B precursor | 99% | 2e-46 | Peptidase_C1 superfamily |
| Cr-CatB7 | XP_002404474 | *Ixodes scapularis* | Cathepsin B endopeptidase | 60% | 4e-94 | Peptidase_C1 superfamily |
| Cathepsin-family D | | | | | | |
| Cr-CatD1 | ACV53024 | *Homarus americanus]* | cathepsin D2 | 51% | 3e-87 | Pepsin retropepsin/Aspartil proteasa |
| Cr-CatD2 | XP_005098204 | *Aplysia californica* | Lysosomal aspartic protease-like | 53% | 5e-32 | Pepsin retropepsin like |
| Cr-CatD3 | ACV53024 | *Homarus americanus* | Cathepsin D2 | 53% | 3e-50 | Pepsin retropepsin-like |
| Cr-CatD4 | AEC03508 | *Polyrhachis vicina* | Cathepsin-D | 42% | 9e-17 | Pepsin-retropepsin like |
| Cr-CatD5 | Q9DEX3 | *Clupea harengus]* | Cathepsin D precursor | 42% | 1e-50 | Pepsin-retropepsin like |
| Cr-CatD6 | ACO15540 | *Caligus clemensi* | Cathepsin D precursor | 67% | 2e-176 | Pepsin-retropepsin like/ Asp |
| Cathepsin-family F | | | | | | |
| Cr-CatF1 | AFQ01139 | *Chilo suppressalis* | Cathepsin F-like protease | 37% | 1e-25 | Peptidase_C1 superfamily/ Inhibitor_I29 |
| Cr-CatF2 | CDJ26737 | *Tityus serrulatus* | cathepsin F-like cysteine peptidase protein | 37% | 1e-26 | Peptidase_C1 superfamily/ Inhibitor_I29 |
| Cr-CatF3 | ABF18890 | *Lygus lineolaris* | Cathepsin-L-like | 50% | 1e-20 | Peptidase_C1 superfamily |
| Cathepsin-family K | | | | | | |
| Cr-CatK1 | [ACO15154](http://www.ncbi.nlm.nih.gov/protein/225718616?report=genbank&log$=prottop&blast_rank=3&RID=V3SF9ZKH01R) | *Caligus clemensi* | Cathepsin K precursor | 40% | 5e-136 | Inhibitor I29 /Peptidase_C1 superfamily |
| Cathepsin-family L | | | | | | |
| Cr-CatL1 | ACO10357 | *Caligus rogercresseyi* | Cathepsin L precursor | 80% | 0.0 | Peptidase_C1/Inhibitor |
| Cr-CatL2 | [ADM53739.1](http://www.ncbi.nlm.nih.gov/protein/305434754?report=genbank&log$=prottop&blast_rank=1&RID=XG9ZK08H014) | *Lepeophtheirus salmonis* | Cathepsin L precursor | 80% | 5e-127 | Peptidase_C1 |
| Cr-CatL3 | ACO10357 | *Caligus rogercresseyi* | Cathepsin L precursor | 98% | 5e-25 | Peptidase superfamily x2 |
| Cr-CatL4 | ACO15375 | *Caligus clemensi* | CathepsinL1precursor | 77% | 9e-50 | Peptidase superfamily |
| Cr-CatL5 | ACO10357 | *Caligus rogercresseyi* | Cathepsin L precursor | 94% | 2e-47 | Peptidase C1 superfamily |
| Cr-CatL6 | XP_001022365 | *Tetrahymena thermophila* | Papain superfamily cistein protease | 45% | 3e-26 | Peptidase_C1 superfamily/ Inhibitor_I29/Porin3 superfamily |
| Cr-CatL7 | ACO10357 | *Caligus rogercresseyi* | Cathepsin L precursor | 90% | 2e-49 | Peptidase_C1 superfamily |
| Cr-CatL8 | EWS74459 | *Tetrahymena thermophila* | Papain family cysteine protease | 46% | 2e-36 | Peptidase_C1 superfamily |
| Cr-CatL9 | CBJ31444 | *Ectocarpus siliculosus]* | Cathepsin L-like proteinase | 41% | 2e-27 | Peptidase_C1 superfamily/ Inhibitor_I29 |
| Cr-CatL10 | EJY65772 | *Oxytricha trifallax* | Cathepsin L | 55% | 4e-40 | Peptidase_C1 superfamily |
| Cr-CatL11 | NP_001163996 | *Tribolium castaneum* | Cathepsin L-like proteinase precursor | 57% | 2e-22 | Peptidase_C1 superfamily |
| Cr-CatL12 | ABQ23400 | *Trypanoplasma borreli* | Cathepsin L isotype 3 | 47% | 8e-26 | Peptidase_C1 superfamily |
| Cr-CatL13 | ACO10357 | *Caligus rogercresseyi* | Cathepsin L precursor | 86% | 8e-32 | Peptidase_C1 superfamily |
| Cr-CatL14 | ABS17682 | *Artemia salina* | Cathepsin L-1 | 47% | 7e-38 | Peptidase_C1 superfamily |
| Cr-CatL15 | EWS74459 | *Tetrahymena thermophila* | Papain family cysteine protease | 46% | 2e-17 | Peptidase_C1 superfamily |
| Cr-CatL16 | ACO10357 | *Caligus rogercressey* | Cathepsin L precursor | 69% | 2e-42 | Peptidase_C1 superfamily |
| Cr-CatL17 | XP_004341646 | *Acanthamoeba castellanii* | Papain family cysteine protease | 49% | 3e-42 | Peptidase_C1 superfamily |
| Cr-CatL18 | EJY83513 | *Oxytricha trifallax* | Cathepsin L | 55% | 5e-69 | Peptidase_C1 superfamily |
| Cr-CatL19 | AFV73398 | *Haliotis discus hannai* | Cathepsin L | 96% | 3e-92 | Peptidase_C1 superfamily |
| Cr-CatL20 | XP_001013459 | *Tetrahymena thermophila* | Papain family cysteine protease | 42% | 1e-22 | Peptidase_C1 superfamily |
| Cr-CatL21 | XP_004032131 | *Ichthyophthirius multifiliis* | Papain family cysteine protease | 41% | 1e-70 | Peptidase_C1 superfamily |
| Cr-CatL22 | XP_001026313 | *Tetrahymena thermophila* | Papain family cysteine protease containing protein | 47% | 1e-90 | Peptidase_C1 superfamily/Inhibitor I29 |
| Cr-CatL23 | ACO15375 | *Caligus clemensi* | Cathepsin L1 precursor | 65% | 4e-41 | Peptidase_C1 superfamily |
| Cr-CatL24 | ACO15375 | *Caligus clemensi* | Cathepsin L1 precursor | 52% | 3e-36 | Inhibidor I29 |
| Cr-CatL25 | ACO15375 | *Caligus clemensi* | Cathepsin L1 precursor | 65% | 3e-57 | Peptidase_C1 superfamily |
| Cr-CatL26 | ACO14903 | *Caligus clemensi* | Cathepsin L precursor | 61% | 4e-129 | Peptidase_C1 superfamily/ Inhibitor I29 |
| Cr-CatL27 | ACO10357 | *Caligus rogercresseyi* | Cathepsin L precursor | 82% | 2e-142 | Peptidase_C1 superfamily |
| Cathepsin-family S | | | | | | |
| Cr-CatS1 | XP_006755711 | *Myotis davidii* | Cathepsin S isoform X1 | 45% | 6e-16 | Peptidase_C1 superfamily |
| Cathepsin-family Z | | | | | | |
| Cr-CatZ1 | XP_005524892 | *Pseudopodoces humilis* | Cathepsin Z | 53% | 2e-16 | Peptidase_C1 superfamily |
| Cr-CatZ2 | XP_008118699 | *Anolis carolinensis* | Cathepsin Z-like | 57% | 1e-22 | Peptidase_C1 superfamily |
| Cr-CatZ3 | ADD38893 | *Lepeophtheirus salmonis* | Cathepsin Z | 43% | 4e-12 | Peptidase_C1 superfamily |
| Cr-CatZ4 | XP_001471259 | *Tetrahymena thermophila]* | Cathepsin Z | 55% | 1e-87 | Peptidase_C1 superfamily |
| Cr-CatZ5 | EJY71046 | *Oxytricha trifallax* | Papain family cysteine protease | 55% | 7e-22 | Peptidase_C1 superfamily |
| Cr-CatZ6 | XP_004555430 | *Maylandia zebra* | Cathepsin Z-like | 77% | 1e-46 | Peptidase_C1 superfamily |
| Cr-CatZ7 | XP_004555430 | *Maylandia zebra* | Cathepsin Z-like | 77% | 1e-46 | Peptidase_C1 superfamily |
| Cr-CatZ8 | EWM25400 | *Nannochloropsis gaditana* | Cathepsin z | 50% | 2e-55 | Peptidase_C1 superfamily |
| Cr-CatZ9 | XP_001471259 | *Tetrahymena thermophila* | Cathepsin z | 61% | 3e-76 | Peptidase_C1 superfamily |
| Cr-CatZ10 | ADD38893 | *Lepeophtheirus salmonis* | Cathepsin Z | 86% | 8e-174 | Pepidase_C1 /Peptidase_C1 superfamily |

**Table S1B**. Sequences of primers used in this study for cathepsin sequence.

| ID | Sequence (5'-3') | T° Melting |
| --- | --- | --- |
| Cr-CatL2_F | TATACACAATGTCACTAC | 45°C |
| Cr-CatL2_R | AAGTATAGGGATATTCTTC |  |
